# Machine Learning Model as a Useful Tool for Prediction of Thyroid Nodules Histology, Aggressiveness and Treatment-Related Complications

**DOI:** 10.3390/jpm13111615

**Published:** 2023-11-17

**Authors:** Valeria Dell’Era, Alan Perotti, Michele Starnini, Massimo Campagnoli, Maria Silvia Rosa, Irene Saino, Paolo Aluffi Valletti, Massimiliano Garzaro

**Affiliations:** 1 ENT Division, Novara Maggiore Hospital, 28100 Novara, Italy; mariasilvia.rosa@maggioreosp.novara.it (M.S.R.); 20017512@studenti.uniupo.it (I.S.); 2 CENTAI Institute, 10138 Turin, Italy; alan.perotti@centai.eu (A.P.);; 3 Departament de Fisica, Universitat Politecnica de Catalunya, Campus Nord, 08034 Barcelona, Spain; 4 Department of Otorhinolaryngology, Ss. Trinità Hospital, 28021 Borgomanero, Italy; massimo.campagnoli@asl.novara.it; 5 ENT Division, Health Science Department, School of Medicine, Universitá del Piemonte Orientale, 28100 Novara, Italy; paolo.aluffi@med.uniupo.it (P.A.V.); massimiliano.garzaro@uniupo.it (M.G.)

**Keywords:** thyroid cancer, machine learning, surgical approach, surgical complication

## Abstract

Thyroid nodules are very common, 5–15% of which are malignant. Despite the low mortality rate of well-differentiated thyroid cancer, some variants may behave aggressively, making nodule differentiation mandatory. Ultrasound and fine-needle aspiration biopsy are simple, safe, cost-effective and accurate diagnostic tools, but have some potential limits. Recently, machine learning (ML) approaches have been successfully applied to healthcare datasets to predict the outcomes of surgical procedures. The aim of this work is the application of ML to predict tumor histology (HIS), aggressiveness and post-surgical complications in thyroid patients. This retrospective study was conducted at the ENT Division of Eastern Piedmont University, Novara (Italy), and reported data about 1218 patients who underwent surgery between January 2006 and December 2018. For each patient, general information, HIS and outcomes are reported. For each prediction task, we trained ML models on pre-surgery features alone as well as on both pre- and post-surgery data. The ML pipeline included data cleaning, oversampling to deal with unbalanced datasets and exploration of hyper-parameter space for random forest models, testing their stability and ranking feature importance. The main results are (i) the construction of a rich, hand-curated, open dataset including pre- and post-surgery features (ii) the development of accurate yet explainable ML models. Results highlight pre-screening as the most important feature to predict HIS and aggressiveness, and that, in our population, having an out-of-range (Low) fT3 dosage at pre-operative examination is strongly associated with a higher aggressiveness of the disease. Our work shows how ML models can find patterns in thyroid patient data and could support clinicians to refine diagnostic tools and improve their accuracy.

## 1. Introduction

Thyroid carcinomas are the most common endocrine cancers and are usually associated with good survival. Their incidence and mortality trends have been identified as being consistent with over-diagnosis, and several recent efforts have been made to mitigate this problem [1]. Despite the usual good prognosis, some variants may appear more aggressive than other, influencing the mortality rate. The aggressive behavior has been ascribed to the histologic subtype and/or to the clinic-pathologic features, an issue that remains controversial [2].

Potential “aggressive variables” for consideration include the specific histology (well-differentiated thyroid cancer versus poorly differentiated thyroid cancer), molecular profile, size and location of distant metastases (pulmonary metastases versus bone metastases versus brain metastases), functional status of the metastases (RAI avid versus 18FDG-PET avid) and effectiveness of initial therapy (completeness of resection, effectiveness of RAI, external beam radiation or other systemic therapies) [3].

Fine needle aspiration cytology (FNAC) is a simple, safe, cost-effective and accurate diagnostic tool for the initial screening of patients with thyroid nodules, but the recent literature data has shown some possible limits [4]. False negatives are not so rare and should be related to sampling error (the size and number of nodules lead to heterogeneity and unsampled areas), while the majority of false-positive diagnoses are related to interpretative errors due, for example, to overlapping cytological features in adenomatous hyperplasia, thyroiditis and cystic lesions. 

For these reasons, it is of fundamental importance to match the FNAC result with a series of other clinical and anamnestic data, in order to obtain adequate diagnostic sensitivity and specificities. In this regard, the American Thyroid Association recommended that serum thyrotropin (TSH) should be measured during the initial evaluation of a patient with a thyroid nodule; FNAC is the procedure of choice in the evaluation of thyroid nodules, and it is recommended for nodules > or = 1 cm in greatest dimension with high or intermediate suspicion sonographic pattern. Sonographic patterns with an estimated high risk of malignancy are solid hypoechoic nodules or solid hypoechoic components of a partially cystic nodule with one or more of the following features: irregular margins (infiltrative, micro-lobulated), microcalcifications, a taller-than-wide shape, rim calcifications with small extrusive soft tissue component, evidence of extra thyroidal extension (ETE). Sonographic patterns with estimated intermediate risk of malignancy are hypoechoic solid nodules with smooth margins and without microcalcifications, ETE, or a taller-than-wide shape [3].

Therefore, what the doctor must do when visiting a patient suffering from a thyroid nodule is combine all these variables and formulate his own suspicion of risk. If this process were infallible and repeatable, with high sensitivity and specificity, few misdiagnoses would be made.

For these purposes (containing over-diagnosis, predicting aggressive variants and refining diagnostic tools), a machine learning (ML) approach could offer the opportunity to stratify patients in risk classes and consequently to perform a more accurate diagnosis and therapy. 

Recently, ML approaches have been successfully applied to healthcare datasets. However, these models often behave as black boxes and do not allow for clinical interpretation of results [5,6,7,8].

Contemporary clinical trials have shown that an artificial intelligence model’s performance matched that of experienced radiologists and pathologists [8]. Elliott Range DD et al. reported that the performance of the ML in predicting thyroid malignancy with FNAC is comparable to the performance of an expert cytopathologist, suggesting that matching ML and medical diagnoses can offer better performance than either alone [6].

The aim of this work is the application of ML to predict tumor histology (HIS), aggressiveness and post-surgical complications in a population of consecutive patients who underwent thyroid surgery in a single center during a 13-year period.

## 2. Materials and Methods

### 2.1. Data Collection

This retrospective study was conducted at a single academic center between January 2006 and December 2018 and received approval by the ethics committee of Maggiore Hospital (CEI 133/2022). We reviewed data about 1218 patients who underwent surgery at the ENT Division of Eastern Piedmont University, Novara (Italy). Informed consent was obtained from all subjects involved in the study. Data analysis includes only primitive thyroid disease. Thyroidectomy performed during total laryngectomy, parathyroidectomy or other major surgery are not included. Patients were excluded if their medical records were not available or missing. For each patient, the following data were collected: general information (sex, age, anthropometric data), clinical history (smoke, alcohol, radiation, comorbidity), thyroidal specific diseases (and their treatment), surgical options (type of resection, days of hospitalization, complications). Partial thyroidectomy “PT” (e.g., resection of one lobe +/− isthmus) was performed just in case of known or suspected monolateral benign disease. Total Thyroidectomy (TT), Near total thyroidectomy (NTT) and Sub-Total thyroidectomy (STT) were indicated in case of malignancy or symptomatic bilateral benign disease. In all patients, an external median cervical approach allowed the surgical excision with the purpose of identifying and preserving recurrent laryngeal nerves (RLN) and parathyroid glands. Peri-operative management includes the placement of a drainage tube (Jackson Pratt drainage with inner diameter about 2.2 mm), intravenous antibiotic prophylaxis (Cefamandole 2 gr) and accurate hemostasis with bipolar forceps, absorbable sutures and absorbable hemostatic devices like fibrillary or hemostatic sponges. All surgical complications were recorded, both general (hemorrhage, hematoma, other neck swelling, infection of surgical site) and specific to thyroidal surgery (hypocalcemia, recurrent laryngeal nerve palsy and less common external branches of the superior laryngeal nerve injury, esophageal lesion, tracheal perforation, subcutaneous emphysema, thoracic duct injury, cervical sympathetic nerve chain lesion). Serum calcium levels at 6, 24, 48, 72 and 96 h after surgery were recorded, as well as clinical signs and symptoms of hypocalcemia. Transient hypocalcemia (tHypoCa) was defined as serum calcium level at discharge < or = 8.0 mg/dL with the necessity of calcium supplementation for less than 6 months after surgery. Transient recurrent laryngeal nerve palsy (tRLNP) is defined as hypomobility or paralysis of one or both vocal folds lasting less than 6 months after surgery. Hypocalcemia and inferior laryngeal nerve palsy are considered as permanent if still present 6 months after surgery. 

The diagnosis (benign or malignant) and aggressiveness of the tumors were determined by pathological evaluation of thyroidectomy specimens. In particular, aggressiveness was assessed according to the American Thyroid Association (ATA) 2015 risk stratification system for differentiating thyroid carcinoma [3].

### 2.2. Data Cleaning

Features with more than one-third of values missing have been discarded. For remaining features, missing data for numeric variables was handled using median imputation. Missing categorical data was assigned a value of “Unknown”. In order to reduce data sparsity, we added binary variables (YES/NO) to resume several multiclass categorical or numeric features. For example, we generated a derivate feature, “DIAGNOSTIC PRE”, including ultra-sound (US) data, fine-needle ago-biopsy (FNAB) results and clinical presentation, according to American Thyroid Academy (ATA) guidelines, in order to divide thyroid nodules into suspected (“YES”) or not (“NO”) for malignancy. We classified as “affected by malignancy” a patient whose specimen contained thyroid cancer cells. Lymph nodes were positive if confirmed by histo-pathological exam. 

Other examples of derivate features are: “ECO YES/NO” on the basis of suspicious US patterns (micro-calcification, vascularization, irregular margin, solid composition, hypo-echogenicity, elongated shape) or “AGGRESSIVENESS YES/NO” according to the presence/absence of ATA suggested criteria (aggressive pathological subtypes like tall cell, columnar cell or hobnail, extra-thyroidal extension, lymph node involvement and distant metastasis). The final dataset is composed by n = 1218 patients, described by 95 features, divided into pre-surgery (46) and post-surgery (49) based on the fact that they described characteristics well known before surgery or revealed after the procedure. For example, SEX, AGE, cytological results, and US features were included as pre-surgery variables; capsular invasion, malignancy, complication and serum post-operative blood calcium levels were included as post-surgery characteristics.

### 2.3. Prediction Tasks

We aim to predict two main events: (i) the tumor histology and its aggressiveness, including T and N YES/NO variables, (ii) complications, including transient hypocalcemia and duration of post-operative recovery. Since predicting the exact duration of the post-operative recovery would be unfeasible, we aim to predict if the post-operative recovery will be longer than three days (we always attached a drainage tube, and the third day usually corresponded with drainage removal time). Each event prediction is estimated by using two different sets of features: all features and only pre-surgery features. Given that post-surgery features are highly informative of the surgery outcome, we expect prediction accuracy to drop when using only pre-surgery features. For each prediction task, we exclude variables strictly related to target prediction, e.g., we exclude all calcium measurements after surgery when predicting transient hypocalcemia.

### 2.4. Class Re-Balancing

**Figure 1:**
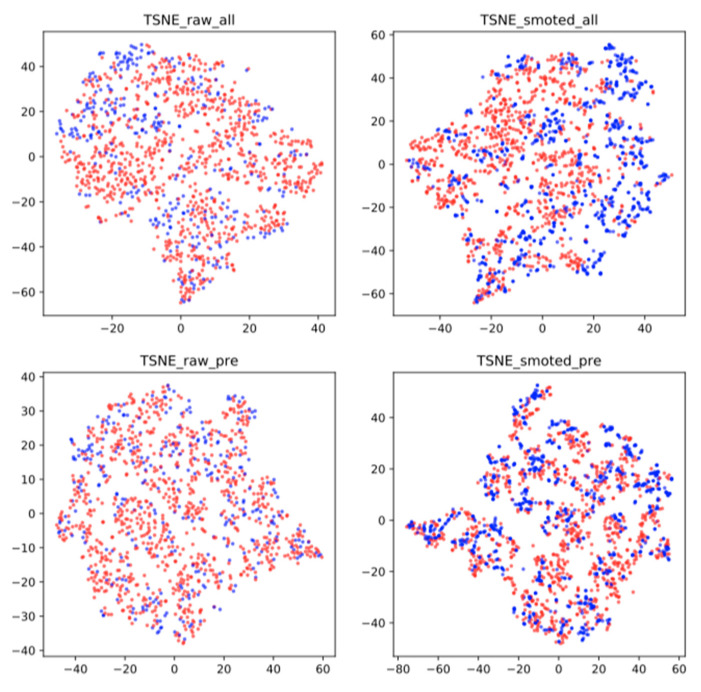
T-distributed Stochastic Neighbor Embedding (t-SNE) visualization of data points by considering all variables (**top** row) or only pre-surgery variables (**bottom** row), for the original dataset (**left** column) and data oversampled by SMOTE algorithm, (**right** column). Blue (red) data points represent patients with malign (benign) tumor histology.

Classes are naturally unbalanced in several prediction tasks, especially for complications such as hypocalcemia, since few patients usually experience it. For example, only 30 patients out of 1200 experienced permanent complications related to vocal cords, 72 patients experienced permanent hypocalcemia and 37 patients had bleeding. Since most machine learning algorithms perform badly with such strongly unbalanced classes, we will apply the Synthetic Minority Oversampling Technique (SMOTE) to oversample the minor class in the training data. The challenge is represented by a minority class that has typically very little data and is often the focus of attention. One approach for handling imbalance is to generate extra data from the minority class, to overcome its shortage of data. Figure 1 shows a t-SNE visualization of the dataset to illustrate how SMOTE works. The minority class—patients with malign tumor histology (represented in blue)—is oversampled by generating synthetic data points. These synthetic patients have features close, in feature space, to the ones of real patients with malign tumor histology.

### 2.5. Model Training

We split the dataset using a 75:25 distribution. Models have been trained with 75% of data, and tested with the remaining 25%. Note that we split data into training and test sets before applying SMOTE to avoid overfitting. Indeed, if SMOTE is applied before the train-test split, some synthetic data points in the test set may be generated from real data points in the training set, yielding a data leak from train to test set and thus overfitting. We compared three off-the-shelf classifiers as provided by scikit-learn: Random Forest, Multilayer Perceptron and k-Nearest Neighbors. Since the performances of these models were similar, we focused on explainable models to understand feature importance. We thus trained the Random Forest model using 3-fold cross-validation. The hyper-parameters were tuned via cross-validated grid search over the number of trees and a maximum tree depth.

## 3. Results

Since the test sets can be strongly unbalanced, prediction tasks are evaluated by using balanced accuracy, defined as the average of recall obtained for each class. Furthermore, given the small size of the data, we check the stability of the classifier by splitting the data into train and test sets 10 times, and then computing the standard deviation σ of the balanced accuracy.

### 3.1. Tumor Histology, Aggressiveness and T/N

The prediction of tumor histology and aggressiveness is accurate (more than 90%), with small drop (4–5%) when using only pre-surgery feats. Prediction of T/N is very accurate (more than 95%) with all features, while we observe a 10% drop in accuracy and some false negatives when using only pre-surgery feats. Pre-surgical features are suggested to be incomplete in predicting cases of occult metastasis in the recurrent level and capsular rupture even in the presence of small thyroid nodes (on which depend the T and N stages).

**Figure 2:**
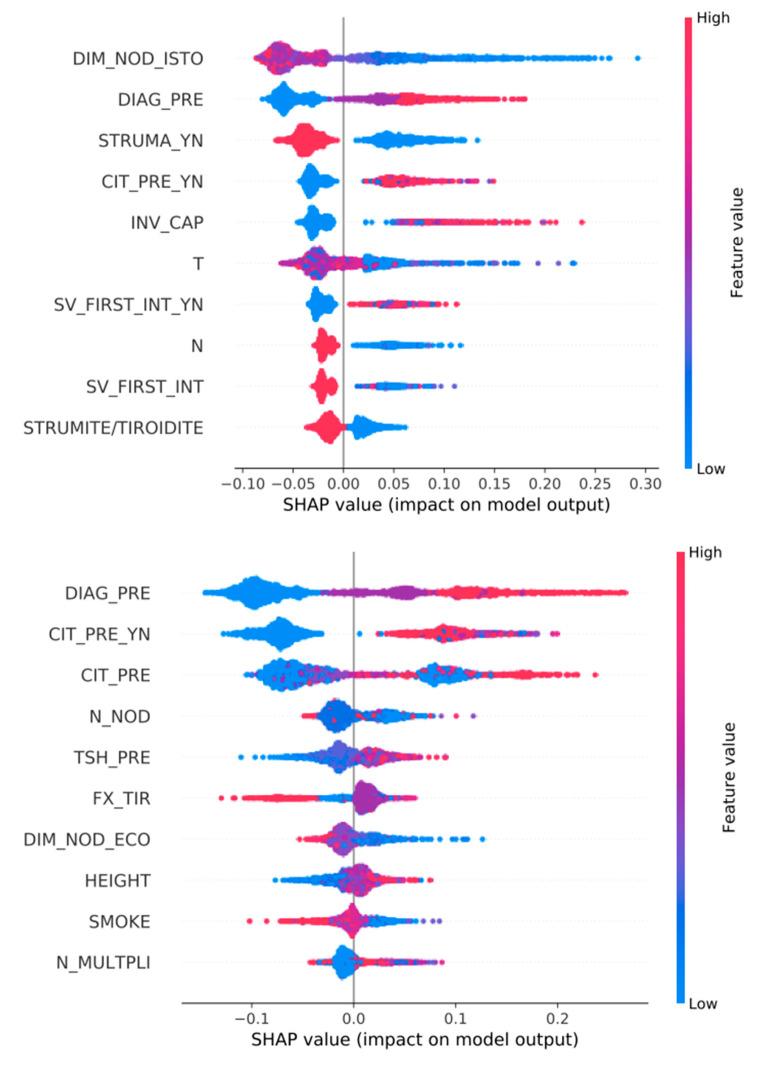
Top 10 most important features for the prediction of tumor HISTOLOGY. Classifiers are trained over all variables (**top**) or only pre-surgery variables (**bottom**).

**Figure 3:**
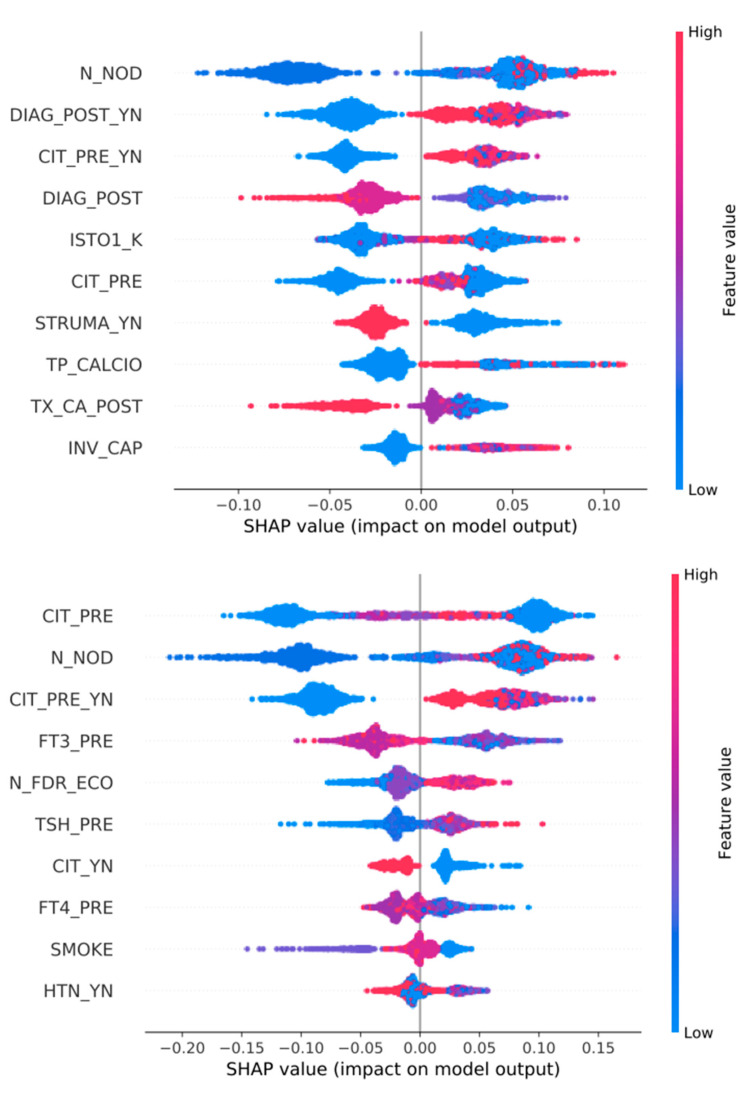
Top 10 most important features for the prediction of tumor AGGRESSIVENESS. Classifiers are trained over all variables (**top**) or only pre-surgery variables (**bottom**).

**Figure 4:**
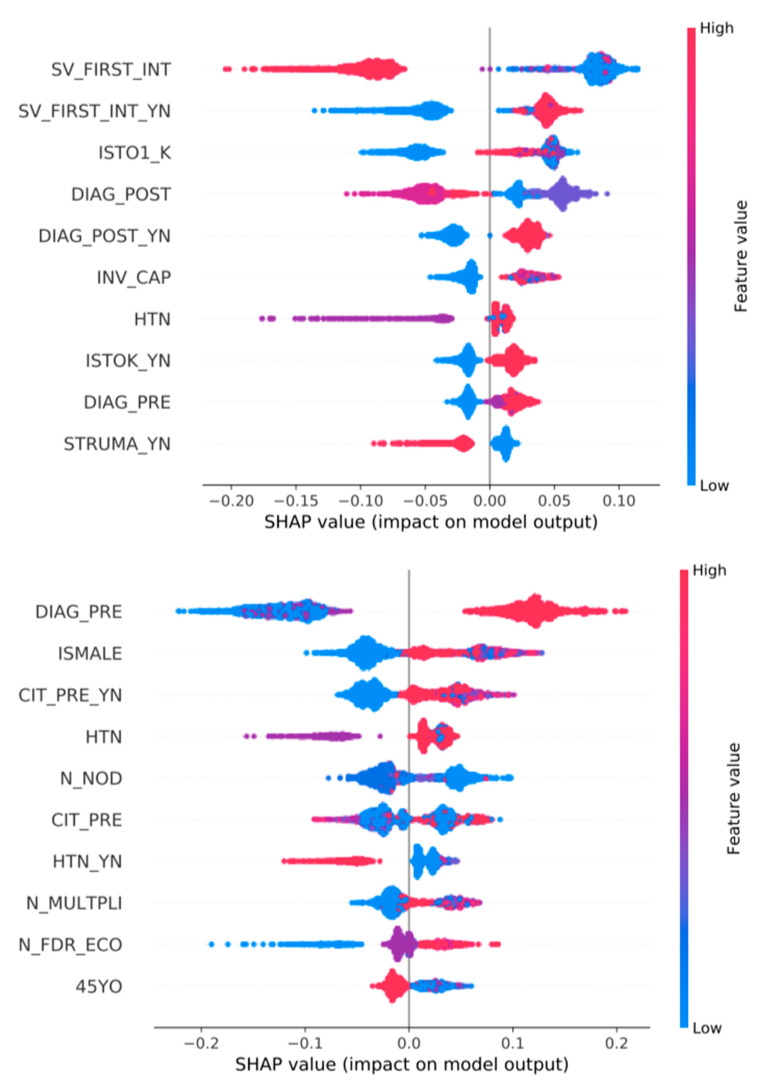
Top 10 most important features for the prediction of variable N (lymph nodes metastasis). Classifiers are trained over all variables (**top**) or only pre-surgery variables (**bottom**).

See Figure 2, Figure 3 and Figure 4.

### 3.2. Transient Hypocalcemia, Complications and Post-Surgery Recovery

Prediction of transient hypocalcemia and complications are nearly accurate (more than 80%), with no drop when using only pre-surgery feats. This means that post-surgery features are not predictive of complications. We observe some false negatives.

**Figure 5:**
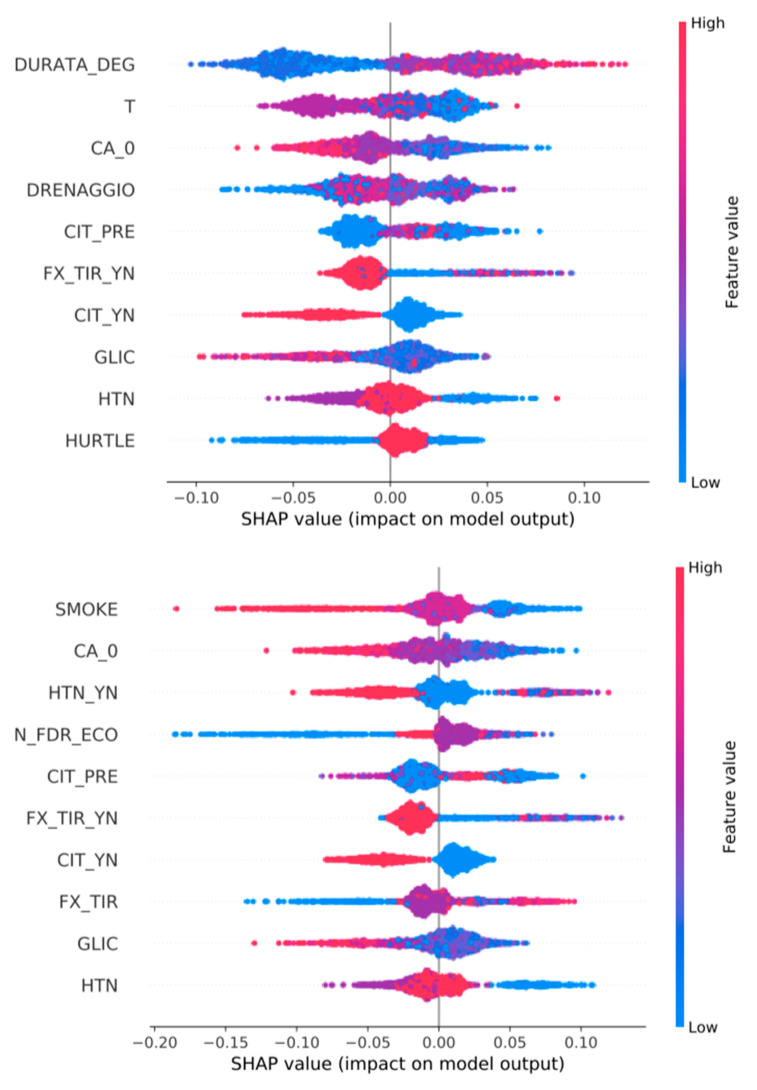
Top 10 most important features for the prediction of TRANSIENT HYPOCALCEMIA. Classifiers are trained over all variables (**top**) or only pre-surgery variables (**bottom**).

**Figure 6:**
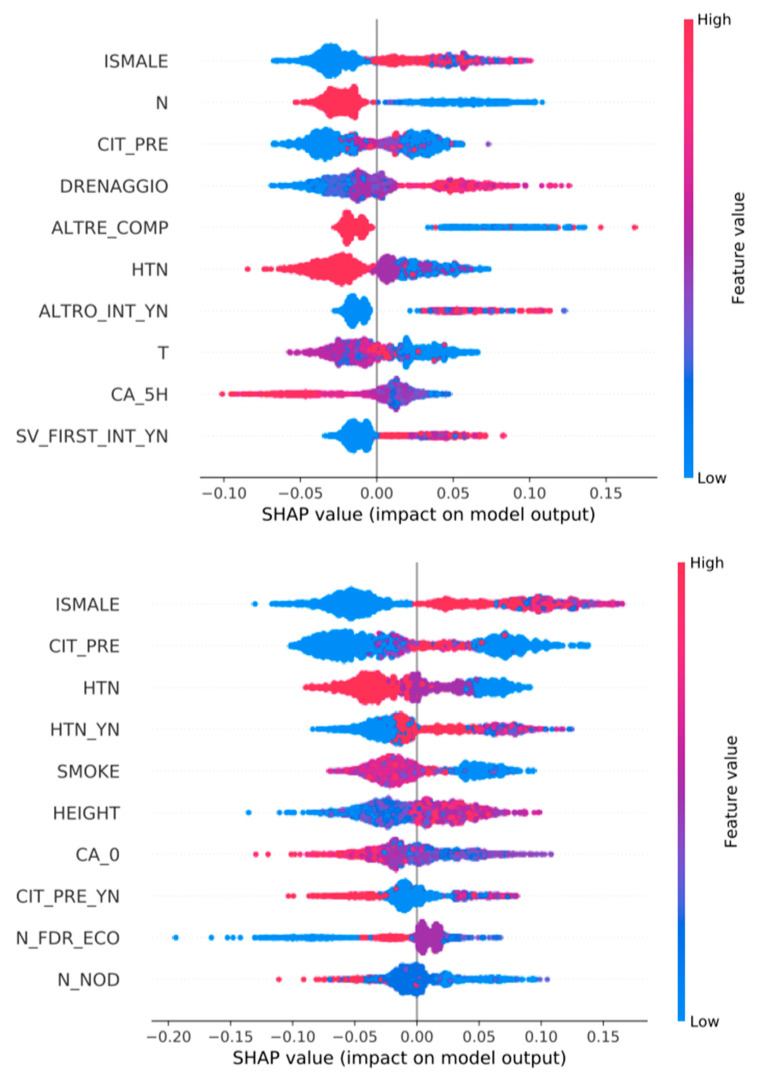
Top 10 most important features for the prediction of COMPLICATIONS (one of permanent hypocalcemia, bleeding or vocal cords permanent disfunction). Classifiers are trained over all variables (**top**) or only pre-surgery variables (**bottom**).

See Figure 5 and Figure 6.

Prediction of the duration of post-surgery recovery is accurate (more than 90%) with all features, while we observe a 10% drop in accuracy and few false negatives and positives when using only pre-surgery feats.

### 3.3. Feature Importance (Figure 2, Figure 3, Figure 4, Figure 5 and Figure 6)

We show all plots, ranking most important features. In each plot, for each feature, each point represents a patient. The color of each point represents the value of the feature for this patient: low (high) value corresponds to blue (red). The importance of the feature in the prediction task is represented by the Shapley Additive Explanations (SHAP) value on the x-axis: patients with positive (negative) impact on the prediction task stay on the right (left) side.

The mentioned variables are defined in Appendix A.

For example, in Figure 6, top row, sex is the most important feature for predicting complications, when using both all features and only pre-surgery features. Male patients have a low value for the sex feature, equal to 1 (blue), while females are equal to 2 (red). Male patients have a positive impact (they are on the right side) on the prediction of complications. That is, male patients are more likely to have complications (one of permanent hypocalcemia, bleeding or vocal cords permanent disfunction). 

Another example is cytology. Patients who had cytology screening are represented in red, while patients who did not have cytology are represented in blue. All red points in the Figure 2, patients who had cytology, have positive impact on prediction of malignant histology. Therefore, patients who underwent cytology screening showed more frequent malignant tumors.

### 3.4. Confusion Matrix

**Figure 7:**
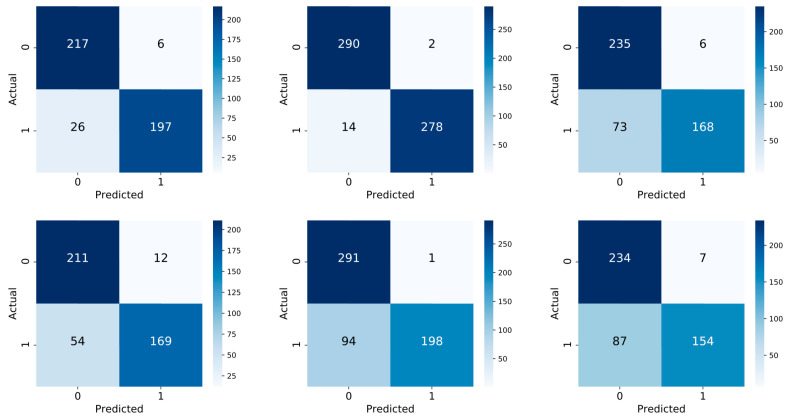
Confusion matrix for the prediction of tumor histology (**left** column), N (**central** column) and transient hypocalcemia (**right** column). Classifier are trained over all variables (**top** row) or only pre-surgery variables (**below** row).

In Figure 7 is represented the confusion matrix that show the performance of our algorithm.

Each row of the matrix represents the instances in a predicted class, while each column represents the instances in an actual class. The name stems from the fact that it makes it easy to see whether the system is confusing two classes (i.e., commonly mislabeling one as another).

Pre-surgery variables showed globally high sensibility and specificity in histology prediction, whereas, concerning nodal involvement and hypocalcemia prediction, specificity of such variables significantly decreased.

## 4. Discussion

The present study confirmed that ML models can successfully help clinicians to improve diagnostic accuracy. First, a data set was drawn up, in as much detail as possible, to better describe patients with thyroid disease. Subsequently the characteristics of the population were divided into pre- and post- surgical, in order to identify new characteristics which, combined with those already known in the literature, can increase the diagnostic accuracy with the goal to maximize resection of malignant nodules and mostly minimize resection of benign nodules. 

In the recent literature, some authors, like Guo et al., purposed a robust prediction model on 2423 patients, based on blood parameters (lymphocytes, platelets count, neutrophils, RDW and RDW-CV, PTH and alkaline phosphatase), mixed with BRAFV600E mutation research and clinical features such as gender and age. The obtained results (AUC of 0.874–95%CI, 0.841, 0.906) seem to show a high value in diagnosing benign and malignant thyroid tumors; the limitations relate to the fact that the population belongs to a single region and the absence of correlation with clinical or radiological data [9].

Other previous studies [5] suggested to analyze ultrasound data with an ML approach. As reported by Ha et al., many studies using the ML technique in thyroid imaging have developed Computer-Aided Diagnosis (CAD) systems based on US features, such as composition, shape, margin, echogenicity and calcifications, and have demonstrated their potential in thyroid cancer diagnosis [10].

Zaho et al. presented their personal results which indicate that an approach based on the knowledge of experienced radiologists and the ML classifier can significantly outperform the radiomics approaches and the current biopsy guideline method in terms of diagnosing thyroid nodules and reducing the unnecessary FNAB rate of thyroid nodules. Due to the retrospective nature of the study, the authors encourage further multicenter and prospective studies with long-term follow-up in order to validate such promising results. The ML method has significant potential for enhancing the ability of radiologists to determine the optimal clinical management of thyroid nodules [11].

In a recent review by Ludwig et al., 930 papers published from 2018 to 2022 were analyzed, in order to focus AI innovations in the field of ultrasonography and microscopic diagnosis of thyroid nodules. The authors suggest significant benefits of using CAD systems in diagnosing thyroid nodules, especially for less experienced radiologists, contributing to significantly reducing the inessential FNAB; nevertheless, the benefit of AI in assisting more experienced clinicians still remains an unmet issue [12].

Considering the cytopathology point of view, in 2023, Wong et al. published an update on the current status of ML applied to pathology diagnosis: the recent development of machine learning algorithms will enable cytopathologists to focus their attention on the regions of interest (ROIs), allowing more accurate and faster interpretations.

Future ML algorithms may integrate cytopathology, radiology and clinical information, creating an even more powerful and promising tool in thyroid cancer diagnostic [13].

Among the most recent studies that involve ML and clinical data in order to improve the diagnosis of thyroid cancer, that by Xi et al. analyzed 724 patients with 1232 nodules, creating a data set with age, gender, blood thyroid function examination, ultrasound findings (9 characteristics), laterality and histological results; the authors confirmed that already-known data, such as calcification, large size, cystic composition and enriched blood flow at US, are strong indicators of malignancy. Moreover, the unilaterality seems to be the worst prognostic factor; they obviously concluded that a larger ML model, involving different studies, could be a high-quality dataset for further improvements in predicting thyroid nodule malignancies [14].

As shown in Table 1, the pre-surgical features are the most accurate in predicting histology, aggressiveness, staging and the onset of complications related to surgical treatment. If, in predicting histology, the pre-surgical variables involved are those known in the literature (especially FNAB, ultrasound-derived data and thyroid function), it is interesting to observe that in our population, having an out-of-range (LOW) fT3 dosage at pre-operative examination is strongly associated with a higher aggressiveness of the disease (Figure 3); this trend seems to be confirmed using all variables. This could prove to be very important, considering that, nowadays, tumor aggressiveness, especially of papillary histology, is explicitly based on histological characteristics [8].

Another interesting occurrence, as shown in Figure 5; Figure 6, seems to be that hypertensive patients have a higher incidence rate of transient hypocalcemia and of complications in general (including hemorrhage and recurrent laryngeal nerve palsy). This finding could be related to an uneasy intra-operative control of blood pressure and a higher risk of bleeding, making dissection more difficult.

To the best of our knowledge, the current study presents an original ML model that could be used to evaluate all features describing patients with thyroidal disease, highlighting some clinical variables, that could be related to more aggressive cancer or possible complicated surgery.

Our study has some limitations: First of all, the entire population refers to a single center and is mostly representative of a single region. Moreover, the retrospective nature of the analysis is obviously influenced by some missing data not recorded at surgery time; a prospective dataset collection will significantly improve the strength of the research.

It would also be interesting to compare the predictive model of this ML model with the sensitivity of the assessment that the clinic derives from the combination of the data at his disposal (which of the two has the greater sensitivity in predicting histology, complications, etc.).

## 5. Conclusions

ML algorithms analyzing pre-surgical features may provide a cost-effective and rapid point-of-care addition to the armamentarium of the endocrine surgeon. 

Future studies, including prospective and multicentric analyses, mixing clinical, laboratory and US data, are needed to understand the potential clinical implications of the ML approach in this field.

## Figures and Tables

**Table 1:** Balance accuracy for different prediction tasks.

Prediction Task	All Feats		Pre-Surgery Feats	
	Accuracy	σ	Accuracy	σ
Histology	0.93	0.02	0.88	0.01
Aggressiveness	0.90	0.03	0.86	0.03
T (YES/NO)	0.96	0.01	0.84	0.02
N (YES/NO)	0.96	0.04	0.88	0.05
Transient hypocalcemia	0.83	0.01	0.82	0.01
Complications	0.84	0.07	0.84	0.05
Recovery duration	0.92	0.01	0.84	0.03

## Data Availability

Data are contained within the article.

## Appendix A. Definition of the Included Variables

**Variables**	**Definition**
**ALTRE_COMP**	presence (blue)/absence (red) of complications other than hypocalcemia, RLN palsy, hemorrhage
**ALTRO_INT_YN**	other cervical surgery performed before (yes blue, no red)
**CA_0**	serum calcium level before surgery >/= (red) or < (blue) 8.0 mg/dL
**CA_5H**	serum calcium level 5 h after surgery >/= (red) or < (blue) 8.0 mg/dL
**CIT_YN**	FNAC performed > 1 year before surgery (yes blue, no red)
**CIT_PRE**	FNAC results suggesting benign lesion (blue)/malignancy (red)
**CIT_PRE_YN**	FNAC performed < or = 1 year before surgery (yes red, no blue)
**DIAG_PRE**	clinic, FNAC and US (ultra-sound) suggested malignancy (red) or not (blue)
**DIAG_POST**	benign (red) or malignant (blue) specimen finding
**DIAG_POST_YN**	confirmed malignancy post-surgery (red) or not (blue) (agreement with pre-surgical findings)
**DIM_NOD_ECO**	ultrasound measurement of the biggest nodule >/= 1 cm (blue) or not (red)
**DIM_NOD_ISTO**	pathological measurement of the biggest nodule >/= 1 cm (blue) or not (red)
**DRENAGGIO**	presence of drainage for </= 3 days after surgery (blue) or more (red)
**DURATA_DEG**	discharge </= 3 days after surgery (blue) or more (red)
**FT3_PRE**	serum freeT3 hormone value < or = (blue)/> (red) normal level
**FT4_PRE**	serum freeT4 hormone value < or = (blue)/> (red) normal level
**FX_TIR**	blood thyroid function’s values < or = (blue)/> (red) normal level
**FX_TIR_YN**	blood thyroid function’s tested before surgery (blue) or not (red)
**GLC**	blood glycemia </= (blue) or > (red) normal level (70–100 mg/dL)
**HEIGHT**	taller than median value (red) or not (blue)
**HTN**	drug intake for hypertension (blue) or not (red)
**HTN_YN**	hypertension (blue) or not (red)
**HURTLE**	presence of Hurtle carcinoma (red) or not (blue)
**INV_CAP**	presence (red)/absence (blue) of thyroid capsular invasion (pathological finding)
**ISMALE**	male(blue)/female(red)
**ISTOK_YN**	malignancy confirmed at specimen (red) or not (blue)
**ISTO1_K**	papillar carcinoma confirmed at specimen (red) or not (blue)
**N**	positive lymphonode at specimen (blue) or not (red)
**N_FDR_ECO**	>/= 1 risk factor at ultrasonography (red) or < (blue) [8].
**N_MULTIPLI**	>/= 1 positive (confirmed with FNAC) thyroid nodules (red) or < (blue)
**N_NOD**	1 (red) or > 1 (blue) thyroid nodules
**SMOKE**	active smokers (red) or not (blue)
**STRUMA_YN**	presence (blue)/absence (red) of associated struma at specimen
**STRUMITE/TIROIDITE**	presence (blue)/absence (red) of associated inflammatory infiltration at specimen
**SV_FIRST_INT**	positive lymphonode in recurrent level (blue) or not (red)
**SV_FIRST_INT_YN**	performed dissection of recurrent level (red) or not (blue)
**T**	T stage </=1 (red) or > (blue)
**TP_CALCIO**	oral calcium intake before surgery (blue) or not (red)
**TSH_PRE**	serum TSH hormone value < or = (blue)/> (red) normal level
**TX_CA_POST**	oral calcium intake after surgery (blue) or not (red)
**45YO**	</= 45 years old (blue) or > (red)
